# Emerging Potential of Ras-proximate-1 (Rap1) in Mediating Neurodegenerative Diseases

**DOI:** 10.2174/011570159X440842251020104840

**Published:** 2025-10-31

**Authors:** Yuan Wang, Jun Hu, Qiong Zhu, Shaoyu Wang, Shijia Yu

**Affiliations:** 1 Department of Neurology, Shengjing Hospital, China Medical University, 110004, Shenyang, China

**Keywords:** Neurodegenerative diseases, Rap1, synaptic plasticity, mitochondria, neuroinflammation, oxidative stress

## Abstract

Neurodegenerative diseases have posed a rising global threat to the aging population, presenting structural and functional impairments in the central nervous system. These progressive disorders, which affect the brain and spinal cord, develop due to the continuous loss of neurons and myelin sheaths. Such specific pathophysiological changes lead to neurological dysfunction in Alzheimer's disease, Parkinson's disease, amyotrophic lateral sclerosis, and Huntington's disease, resulting in typical motor dysfunctions and cognitive disorders, as well as symptoms like behavioral abnormalities and personality changes. To date, despite various treatments attempting to manage these symptoms, patients’ quality of life remains severely deteriorated. A few effective therapeutics are available to mitigate the progression of neurodegenerative injuries. Increasing attention is now focused on molecular regulatory mechanisms, particularly the association between immune regulation and the neurovascular unit. A critical component in this process is Ras-proximate-1 (Rap1), a small Guanosine Triphosphatase (GTPase). Rap1 is determined to regulate glia-mediated immunoinflammatory responses, vascular endothelial function, and neuronal activity. It also modulates synaptic plasticity and mitochondrial function *via* autophagy-dependent modulation, which are significantly impacted during neuronal degeneration. Additionally, signaling pathways, including PI3K/Akt and ERK, are identified as its downstream effectors. Furthermore, by mediating the permeability of the blood-brain barrier, Rap1 probably influences neuroimmune-vascular modulation throughout the development of neurological disorders. In this review, we investigate recent studies to explore the emerging therapeutic potential of Rap1 in the inflammation-related regulation within neurodegenerative diseases. We also discuss novel treatments and possible targets, including natural medicines and genetic modulation, to enhance therapeutic effects and improve prognosis.

## 1 INTRODUCTION

Neurodegenerative diseases are the most common Central Nervous System (CNS) injury, characterized by a gradual loss of neurons and progressive neurological dysfunction. Millions of patients worldwide have been affected, resulting in high rates of disability and mortality. As previously reported, the global prevalence of Alzheimer's Disease (AD) has already exceeded fifty million among the aging population [1]. Approximately 2% of individuals over 60 years old are suffering from Parkinson's Disease (PD) around the world [2]. Conversely, Amyotrophic Lateral Sclerosis (ALS) and Huntington's Disease (HD) exhibit relatively low incidence rates but are highly devastating due to their rapid deterioration [3, 4]. A variety of neurological dysfunctionsymptoms arise from the compromise of neuronal and myelin sheath integrity. Typical manifestations include memory loss, personality changes, movement disorders, chorea, and even difficulties in breathing [5]. Pathophysiological features in the brain and spinal cord were exacerbated by disruptions of cellular signaling, protein homeostasis, and mitochondrial function [6]. Moreover, synaptic dysfunctions and mitochondrial disorders lead to interruption of neural repair and regeneration [7]. Recently, evidence indicated that the regulation of neural circuits could mitigate brain injuries [8]. Multiple pharmacotherapies are widely applied to alleviate the discomfort of neurodegenerative diseases. Additional, physiotherapies and diet therapies have gradually garnered increasing attention. Even surgical interventions could be conducted to enhance neural activity in severely affected patients [9]. However, the molecular candidates for early diagnostic and effective treatment remain limited.

To date, growing research has verified that neuroinflammation and oxidative stress act as “double-edged swords” during the progression of neurodegenerative diseases. Activated microglia and astrocytes mediate the innate immunity and profoundly influence the immune microenvironment by releasing inflammatory regulatory factors [10]. As the first responders to injuries in the CNS, microglia are initially conducive to eliminating damaged cells and toxic debris. However, a disturbed immune microenvironment can aggravate neuronal degeneration *via* the sustained release of pro-inflammatory factors such as TNF-α and interleukin-1β (IL-1β) [11]. Correspondingly, the inflammatory attack is compounded by a loss of protective resistance. Synaptic destruction and neuronal injury are induced following the suppression of microglial phagocytosis, which is supposed to clear harmful substances [12]. Additionally, mitochondrial disorders and the loss of dopaminergic neurons are detected in aging patients with weakened immunity [13, 14]. Concurrently, oxidative stress and immuno-inflammatory responses often induce the loss of motor neurons [15]. Furthermore, neuronal dysfunction in the striatum and cortex is observed in patients exhibiting increased expression of interleukin-6 (IL-6) [16, 17]. Regarding the complex crosstalk between the immune system and the CNS, the underlying regulatory mechanisms have become a crucial focus in neurodegenerative diseases research. This intricate crosstalk presents a promising aspect for therapeutic intervention, aiming to simultaneously modulate neural integrity and the dysregulated immune response commonly observed in patients with neurological disorders. Therefore, molecules that act as integrators between the nervous and immune systems are attracting significant attention as novel targets for developing disease-modifying therapies. Ras-proximate-1 (Rap1) is a well-known small Guanosine Triphosphatase (GTPase) within the Ras superfamily. It suggests that Rap1 modulates immune cells’ function and signaling transduction. Similar to other GTPases, Rap1 transits between the active GTP-bound state and the inactive GDP-bound state. This transition is regulated through not only GTPase-Activating Proteins (GAPs) but also Guanine Exchange Factors (GEFs) [18]. Specific GEFs, including C3G, Epac, and RapGEF2, utilize their Ras-Exchange Motif (REM) and homeodomains of Cell Division Cycle 25 (CDC25) to stimulate the activity of Rap1 [19, 20]. Conversely, excision of RapGEF2 can recess the activation of Rap1 and decrease CD41^+^ cell counts by interfering with the B-raf/ERK pathway [21]. Beyond direct activation, Rap1 participates in the regulation of intracellular signaling *via* mediating secondary messengers such as Ca^2+^ and cyclic Adenosine Monophosphate (cAMP) [22-24]. cAMP can facilitate the Epac/Rap1 axis to strengthen the tight junctions between endothelial cells [25]. Once activated, Rap1 interacts with diverse targets, including Raf, B-Raf, and Ral Guanine Nucleotide Dissociation Stimulator (RalGDS), to modulate cellular adhesion, migration, proliferation, and survival [26]. Rap1 also mediates the ERK and cAMP Response Element Binding protein (CREB) signaling pathways, supporting neuronal survival and apoptosis to increase cell viability [27]. Furthermore, both synaptic vesicle trafficking and neurotransmitter releasing are governed by Rap1-coupled Abelson kinase to regulate the synaptic plasticity, during cognitive institution and memory consolidation [28]. Additionally, Rap1 modulates the cytoskeletal proteins to maintain cellular morphology and motility. These dynamic cellular responses could accelerate neural development [29]. Prior studies have already revealed that Methyltransferase-Like 3 (METTL3) could regulate the downstream Rap1 signaling pathway in neurodegenerative diseases through inducing m6A RNA methylation [30]. It is important to clarify the probable molecular mechanisms to provide novel treatment targets. The critical roles of Rap1 in regulating the network between immune responses and CNS are described in Fig. (**1**). In this review, we discuss the emerging potential of Rap1 in mediating pathological progression in neurodegenerative diseases, especially focusing on neuroinflammation, mitochondrial homeostasis, and synaptic plasticity. Latent regulatory measures are analyzed to appropriately equilibrate Rap1 activity in experimental and clinical trials.

## 2 RAP1 IN NERVOUS SYSTEM

Research has shown that Rap1 plays vital roles in regulating neuronal signaling transduction, calcium homeostasis, and cellular stability. Within the CNS, Rap1 modulates the morphology of dendritic spines, the efficiency of synaptic transmission, and synaptic vesicle trafficking [31]. Meanwhile, neurotransmitters, chemokines, growth factors, and adhesion molecules act as mediators during GTPase functioning. Activated Rap1 can induce glial inflammatory responses and microglial polarization, aggravating the destruction of the blood-brain barrier [32]. Previous study indicates that Rap1 signaling cascade is enhanced in the IL-1 driven autoimmune encephalitis through modulating NOD-Like Receptor Family Pyrin domain containing 3 (NLRP3) inflammasomes [33]. Concurrently, cellular polarity and dynamics can be stimulated by the RhoA/Rap1/talin1 axis [34]. Bone Morphogenetic Protein (BMP)-induced signaling is suppressed after Gef26/PDZ-GEF activates Rap1. As reported, increasing Rap1 expression may facilitate the distribution and stability of synaptophysin and synaptotagmin, potentially enhancing synaptic plasticity and resilience [34].

Additionally, plasticity of cellular structure and function is remodeled *via* Rap1-dependent macropinocytosis and presynaptic activity [28]. Since synaptic plasticity contributes to learning and memory potentials, it is characterized by Long-Term Potentiation (LTP) and Long-Term Depression (LTD) [35]. Activated Rap1 can promote integrin-mediated signaling pathways, conducing to the formation and maintenance of functional synapses. Based on previous reports, Epac activates Rap1 to enhance spinal density *via* promoting insertion of α-amino-3-hydroxy-5-methyl-4-isoxazolepropionic acid (AMPA) receptors during LTP [36]. To keep cell viability, up-regulation of Rap1 can stabilize targeted Postsynaptic Density Protein 95 (PSD-95), relieving early synaptic dysfunction and maintaining dendritic morphology [37]. Furthermore, energy production and cellular homeostasis are maintained *via* Rap1-dependent mitochondrial modulation [38, 39]. However, in the early stages of neurodegenerative diseases, neuronal excitotoxicity of glutamate can stimulate the Rap1/ERK pathway, impairing cell activity [40].

**Fig. (2):**
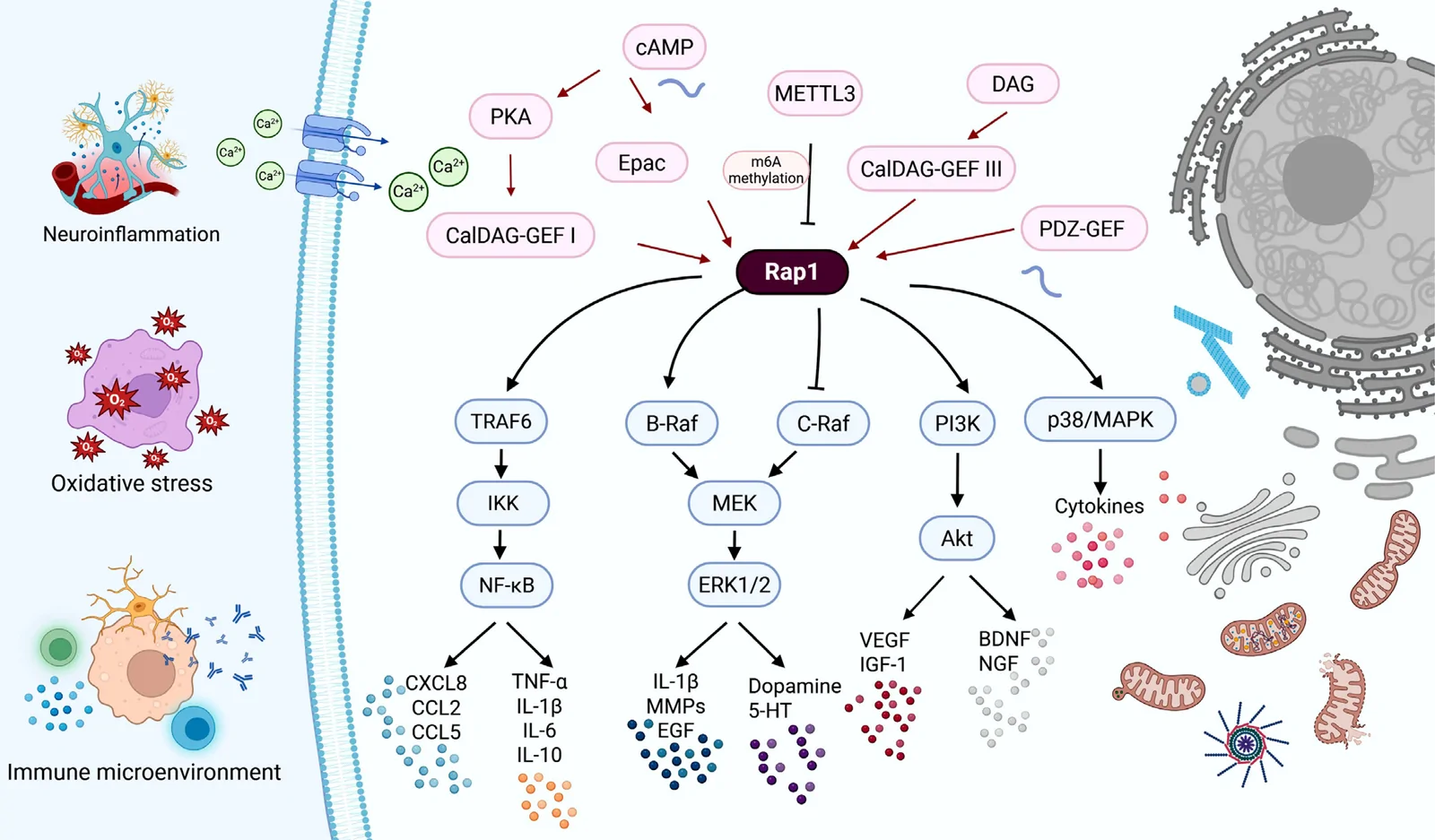
Effects of Rap1 on the nervous system.

Regulators of Rap1 have been reported to affect both the immune microenvironment and neuronal activity. Specific ablation of Rap1 in pyramidal neurons might decrease hyperactivity in stress-associated mental illness by inhibiting threonine phosphorylation [41]. Decreased Rap1 interferes with SH3 and multiple ankyrin repeat domains 2 (SHANK2) localization during synaptic function in hearing regulation [42]. In addition, deletion of Rap1 can promote leptin activity in hypothalamic neurons, resulting in hyperglycemia and overweight [43]. Whereas, lipometabolic disturbance and immune disorders in the nervous system induced by heat stress are mitigated by the Rap1/PI3K/AKT axis [44]. Furthermore, Rap1 accelerates the proliferation and migration of T cells *via* the GTPase-dependent Mitogen-Activated Protein Kinase (MAPK) pathway [45]. Deficiency of GAPs such as Sipa1 and Rasa3 aggravates lymphocyte polarization, disrupting the immune microenvironment [18, 34]. Considering the multiple binding domains of GTPase as scaffolds, IQ Motif Containing GAP1 (IQGAP1) can combine with Rap1, targeting the downstream Rac1 cascade [46]. Similarly, Rap1-GTPases modulate the distribution and abundance of lysosomes *via* Rag and Rheb, restraining activation of the mTORC1 signaling pathway [47]. Interactions among Rap, Rac1, and CDC42 promote actin dynamics, potentially compensating for cytoskeletal deficits. Meanwhile, Rap1 promotes the clearance of abnormal protein aggregation *via* autophagosomes [37]. Given its roles in immune signaling, vascular regulation, and synaptic plasticity, Rap1 emerges as a critical modulator in neurodegenerative disease progression (Fig. **2**).

## 3 ROLES OF RAP1 IN NEUROLOGICAL DISEASES

### 3.1 Alzheimer's Disease (AD)

AD develops typical pathological features, including extracellular accumulation of Aβ plaques and intracellular neurofibrillary tangles composed of hyperphosphorylated tau protein. As listed in Table **1**, evidence has shown that Rap1 influences cellular activity and neuroinflammation throughout AD progression. Specifically, Rap1 prevents neuronal apoptosis by enhancing the expression of the anti-apoptotic factor Bcl-2 while suppressing levels of the pro-apoptotic factor Bax [48]. The PI3K/Akt and ERK pathways are consequently evoked for downstream signaling transmission [49]. Research implies that Rap1 can restrain the release of inflammatory cytokines by adjusting the activity of glial cells [50]. Additionally, Rap1 may inhibit NF-κB signaling to alleviate the microglia-induced inflammatory response. Concurrently, activated Rap1 mediates cellular polarization and Ca^2+^ influx [51]. Additionally, Rap1 regulates endocytosis and vesicle trafficking during Amyloid Precursor Protein (APP) processing in the accumulation of abnormal Aβ plaques [52, 53]. However, the accompanying redox reaction can reduce Rap1 activity, as a negative feedback loop, to damage synaptic plasticity and cellular viability [54]. Activated Rap1 also restrains levels of tau phosphorylation and attenuates its neuronal toxicity in AD by mediating the ERK/MAPK pathways [55].

As reported, synaptic dysfunction can lead to early cognitive decline in AD. Rap1 may promote the stabilization of AMPA and N-methyl-D-aspartate (NMDA) receptors of synapses, which are essential for LTP during the consolidation of learning and memory [56]. In hippocampal neurons, Rap1 activation promotes the formation of dendritic spines and stimulates cytoskeleton remodeling *via* ERK- and B-Raf-dependent synaptic potentiation [57]. In AD mouse, enhancing Rap1 activity might alleviate injuries in synaptic plasticity, while impaired Rap1 signaling accelerates deficits of LTP, together with the loss of spatial memory related to early synaptic failure [50]. Moreover, Rap1 modulates synaptic vesicle cycling and neurotransmitter release to maintain the CNS homeostasis [58]. Small molecules such as miR-32-5p can activate Rap1 to enhance cellular activity throughout the entire stage of AD development, indicating its therapeutic potential in restoring cognitive function [59].

### 3.2 Parkinson's Disease (PD)

In contrast to the cognitive-associated pathology of AD, Parkinson's Disease (PD) is characterized by the progressive loss of dopaminergic neurons in the substantia nigra pars compacta, leading predominantly to motor deficits. Recently, increasing attention has focused on the immune microenvironment and NVUs in the development of PD. In the cortex related to pre-motor PD, the Rap1 signaling pathway is involved in regulating the citrullination network. It reduces the accumulation of α-synuclein induced by inflammatory reaction [60]. Moreover, activation of Rap1 can inhibit pro-apoptotic pathways, defending against oxidative and inflammatory stress in dopaminergic neurons [61]. It protects cell viability in PD *via* the PI3K/Akt and ERK1/2 pathways [62, 63]. While neurotrophin-related signaling, such as the Brain-Derived Neurotrophic Factor (BDNF) pathway, is consequently enhanced for the repair and regeneration of dopaminergic neurons [64].

Furthermore, Rap1 would contribute to removing abnormal protein deposition in PD. Research suggests that Rap1 modulates endosomal-lysosomal trafficking and autophagy pathways during the degradation of α-synuclein. 5-hydroxymethylcytosine can accentuate the toxicity of α-synuclein by targeting Rap1/cAMP and phospholipase D [65]. The formation of Lewy bodies with abnormal α-synuclein is the pathological hallmark of PD, while Rap1 could regulate the aggregation and clearance of the misfolded protein. Disrupted modulation of Rap1 signaling may interrupt autophagic flux, thereby exacerbating oxidative stress and neurotoxicity [66].

In addition, mitochondrial dysfunction is a major cause of energy deficiency, calcium dyshomeostasis, and cell senescence in PD [67]. Reactive Oxygen Species (ROS) can attack mitochondria, causing DNA damage, dynamic disorders, and autophagic dysregulation. Active Rap1 preserves mitochondrial homeostasis by balancing the processes of fission and fusion. In PD models, up-regulated Rap1 can restore mitochondrial membrane potential and improve the biological functions of neurons [67]. Moreover, generation and clearance of mitophagy are modulated by the Rap1-related PKA and AMPK pathways [68]. Therefore, Rap1's significance in PD pivots on its ability to protect vulnerable dopaminergic neurons by mitigating α-synuclein proteotoxicity and sustaining mitochondrial homeostasis, highlighting significant differences from its roles in AD.

### 3.3 Amyotrophic Lateral Sclerosis (ALS)

While Amyotrophic Lateral Sclerosis (ALS) primarily presents with progressive loss of upper and lower motor neurons, Rap1 is identified as a critical mediator in the non-cell autonomous pathology. Rap1 protects motor neuron survival through BDNF and Glial Cell Line-Derived Neurotrophic Factor (GDNF) pathways [69]. Meanwhile, the MAPK/ERK and PI3K/Akt pathways are regulated by Rap1 to reduce apoptosis [48, 56]. Active Rap1 is further verified to promote neuronal outgrowth and prevent axonal degeneration [70, 71]. Recent transcriptomic profiling of spinal cord tissues from ALS patients has identified the downregulated expression of Rap1A and Rap1B, suggesting a potential link between impaired Rap1 signaling and disease progression [72, 73]. However, the exact function of these two isoforms for Rap1 remains unclear.

Notably, Rap1 can modulate the activation and recruitment of microglia. The transcription of pro-inflammatory cytokines like TNF-α, IL-1β, and IL-6 is suppressed following Rap1 activation. Concurrently, reactive gliosis and chronic inflammation are reduced in ALS [74]. Furthermore, Rap1 modulates mitochondrial dynamics and downstream NF-κB signaling, suppressing the destructive cycle of ROS and inflammation that drives motor neuron death [73]. Rap1 can modulate neuron-glia interactions through integrin-mediated signaling and adhesion molecules, thereby influencing immune cell infiltration and microglial polarization [75]. After neuroinflammatory stimulation, Rap1 maintains cytoskeletal integrity and promotes lysosomal vesicle transport [76]. These findings indicate that Rap1 may act as a core balancer during inflammatory responses in ALS.

As is known, oxidative stress is another major threat to the degeneration of motor neurons in ALS. ROS induces dysfunction of mitochondria and glial cells, leading to DNA damage, lipid peroxidation, and protein misfolding. Rap1 can regulate levels of Nicotinamide Adenine Dinucleotide Phosphate (NADPH) to keep mitochondrial dynamics [77]. Activation of Rap1 enhances the expression of Superoxide Dismutase 1 (SOD1), catalase, and glutathione peroxidase *via* the PI3K/Akt and PKA/CREB cascades [78]. Additionally, Rap1 promotes mitochondrial integrity by modulating mitophagy to clear damaged debris [79]. In ALS, therefore, Rap1 emerges as a crucial defender against a hostile microenvironment characterized by exuberant inflammation and oxidative damage, a role that is particularly critical for the survival of the long axons of motor neurons.

### 3.4 Huntington’s Disease (HD)

Huntington's Disease (HD) displays a genetically distinct variation, resulting from a CAG repeat expansion in the huntingtin gene. Mutant Huntingtin (mHTT) forms intracellular aggregates, which interrupt protein homeostasis and trigger neuronal death. Currently, the core challenge is to elucidate the mechanisms underlying the clearance of a specific mutant protein and its downstream transcriptional dysregulation. As reported, Rap1 can alleviate mHTT toxicity by interacting with molecules such as B-Raf and ERK [28, 80]. Besides, Rap1 mediates autophagy, providing an alternative pathway to clear abnormally accumulated proteins. Regarding impaired autophagy as a hallmark of HD, enhanced Rap1 activity can upregulate autophagic flux and reduce aggregated mHTT [28]. Furthermore, Rap1 inhibits disturbed calcium signaling transduction between mHTT, mitochondria, and NMDA receptors. Dysregulated calcium homeostasis can exacerbate mHTT toxicity. Rap1 maintains intracellular calcium levels as a neuroprotector through cAMP-dependent pathways. It is suggested that activated Rap1 can counteract mHTT-induced stress response in neurons [81]. However, it remains unclear whether Rap1 directly influences neuronal resilience.

With regard to synaptic function, loss and disrupted glutamate signaling are known to aggravate cognitive and motor deficits. Rap1 promotes synaptic function and maintains the morphology of dendritic spines *via* impacting AMPA receptor trafficking. Activated Rap1 induces synaptic strengthening during LTP *via* ERK and p38/MAPK pathways [56]. Studies have shown that Rap1 signaling increases spine density, potentially rescuing the loss of dendritic spines induced by mHTT [82, 83]. As is known, corticostriatal synapses exhibit lower plasticity and impaired Excitatory Postsynaptic Currents (EPSCs) due to dysfunctional PSD-95 in HD. Rap1 can stabilize PSD-95 to preserve synaptic integrity [84]. Additionally, Rap1 is involved in restoring the excitatory-inhibitory balance in HD by regulating GABAergic signaling cascades. However, excessively activated Rap1 might deteriorate excitotoxicity by overstimulating glutamate receptors [85].

Moreover, mHTT can restrain the expression of neuronal PAS domain protein 4 (Npas4) *via* energic deficits and oxidative stress. Correspondingly, the activity of transcription factors for SIRT1, including CREB, PGC-1α, and SP1, is decreased. Rap1 may competitively inhibit mHTT binding to CREB-Binding Protein (CBP), suppressing downstream MAPK transcription [86, 87]. By enhancing CREB activity, Rap1 can upregulate the expression of BDNF as a neurotrophin in HD [86]. Rap1 signaling cascades may also upregulate levels of PGC-1α, contributing to mitochondrial function and neuronal survival [27]. Recent studies indicate that Rap1 is involved in epigenetic modifications such as histone acetylation. Elevating Rap1 activity can promote chromatin remodeling, allowing the re-expression of silenced genes in HD [88]. The Rap1 pathway in HD is engaged in promoting clearance of the causative mutant protein to rectify the corresponding transcriptional and epigenetic imbalances. It is crucial to achieve a proper activation of Rap1.

### 3.5 Other Neurological Disorders

Beyond these classic proteinopathic neurodegenerative diseases, Rap1 can influence other neurological alterations where neuroinflammation and vascular integrity are paramount, such as Multiple Sclerosis (MS) and stroke. Since MS is characterized by immune-mediated demyelination and axonal damage, autoreactive T cells and macrophages are activated to infiltrate the CNS. During pathological progression, Rap1 regulates the adhesion and migration of leukocytes across the blood-brain barrier as a core mediator. Activated Rap1 enhances the integrin-mediated adhesion of T cells to endothelial cells, thereby facilitating their infiltration into the CNS [89]. As listed in Table **1**, studies using the experimental autoimmune Encephalomyelitis (EAE) model reveal that inhibiting Rap1 can reduce T-cell extravasation and ameliorate disease severity [90]. Rap1 also interacts with Epac and cAMP pathways to modulate cytokine production, promoting anti-inflammatory IL-10 expression in macrophages [91]. Furthermore, it may contribute to remyelination and neuroprotection, as Oligodendrocyte Precursor Cells (OPCs) differentiated *via* the Rap1-dependent pathway. Activated Rap1 may enhance OPC maturation *via* ERK signaling to promote myelin repair [92]. By regulating AMPA receptor trafficking, Rap1 enhances synaptic plasticity to counteract injuries in MS [56]. However, it is important to note that excessive Rap1 activity in immune cells could exacerbate inflammation, thereby aggravating neurological dysfunction [93].

Stroke disrupts blood-brain barrier integrity, including endothelial cells, tight junctions, and astrocytic end feet, leading to vasogenic edema and neuronal damage. Ischemia-reperfusion injury is confirmed to exacerbate neuronal death through oxidative stress, mitochondrial dysfunction, and excitotoxicity. Rap1 plays a pivotal role in stabilizing endothelial tight junctions and regulating vascular permeability. Studies on endothelial cells demonstrate that Rap1 activation can reduce permeability of the blood-brain barrier under hypoxic conditions [94]. By activating downstream effectors, including afadin and Rac1, Rap1 stabilizes adherens and tight junction proteins, such as Vascular Endothelial cadherin (VE-cadherin) and claudin-5 [25, 95]. Furthermore, the interaction between Rap1 and cAMP-dependent pathways can facilitate tight junction assembly through reducing Matrix Metalloproteinase (MMP)-mediated blood-brain barrier degradation [96, 97]. Rap1 modulates NMDAR-dependent glutamate signaling to prevent neurons from calcium overload. Additionally, Rap1 modulates angiogenesis for long-term recovery. Activation of Rap1 enhances Vascular Endothelial Growth Factor (VEGF) expression and endothelial cell proliferation, facilitating vascular repair around ischemic regions [98]. All the above-mentioned findings verify the significant potential of Rap1 in the management of ischemic stroke.

## 4 DEVELOPMENT OF THERAPEUTICS TARGETING RAP1

The comprehensive analyses of Rap1's roles among different neurological disorders apparently lead to the challenge of how these insights can be conducted therapeutically. Given the crucial and diverse roles of Rap1 in regulating the complex interplay between the immune microenvironment and neurons, it is a pivotal target for designing efficient therapeutic strategies to improve prognosis. The ubiquitous yet “double-edged sword” feature of Rap1 signaling generates not only an opportunity but also a great difficulty for drug development. Recent therapeutic strategies are divided into groups by either enhancing Rap1's neuroprotective functions or inhibiting its detrimental inflammatory effects. As described in Table **2**, several compounds have been identified to modulate Rap1 activity for therapeutic benefits. Glucose-dependent Insulinotropic Polypeptide (GIP) would remit ferroptosis and maintain neuronal homeostasis by mediating the Epac/Rap1 axis [99]. Pde6δ could influence Ca^2+^ influx, decrease neuronal loss, and improve cognitive performance *via* activating Rap1 in transgenic AD mice [51]. Besides, 8-pCPT-2’-O-Me-cAMP, a selective agonist of Epac, enhances Rap1 activity to reduce neuroinflammation and stabilize the blood-brain barrier [32]. The evolution of such compounds from preclinical models to clinical application aims to overcome problems focusing on blood-brain barrier penetrability, target specificity, and isoform-selective effects, which are critical considerations for pharmaceutical development.

In addition, recent research has revealed that natural medicines might mediate Rap1 activity, providing another approach for better prognosis. Lingguizhugan decoction can facilitate Rap1 activity and restrain expression of iNOS, TNF-α, and IL-1β, thereby protecting neuronal viability in AD [100]. Genkwanin from *Rosmarinus officinalis L* binds with SRC genes to target the downstream Rap1 pathway, prohibiting neuronal apoptosis and elevating cellular activity [54]. Cannabidiol might modify Cannabinoid Receptor 1 (CNR1) levels to mediate Rap1 expression, regulating dopaminergic synaptic injury and neuroinflammation in PD [101]. Besides, protocatechuic acids contained in *Sanghuang* could stimulate the VEGF-triggered activation of Rap1, thereby modulating platelet activity and endothelial inflammatory response [102]. Hydroxychavicol is regarded as a candidate to treat AD by mediating Rap1-induced synaptic plasticity and neuronal excitability [103]. Furthermore, marine polyphenols could alleviate neuroinflammation and impact endothelial adhesion *via* the Akt/Rap1 pathway [104]. Inflammation is restrained by leech *via* upregulating Rap1 expression in neurodegenerative diseases [105]. While natural compounds demonstrate multi-targeted potentials, the translation into evidence-based medicines requires more rigorous standardization, pharmacokinetic studies, and validation in substantial clinical trials to establish solid evidence for human use.

Currently, gene-modified therapy presents a promising strategy to overcome the limited abundance of small-molecule modulators by directly upregulating endogenous Rap1 expression. Viral vectors, particularly Adeno-Associated Virus (AAV), have been used to deliver constitutively active Rap1 (Rap1-CA) in preclinical models, resulting in reduced neuronal apoptosis and improved synaptic function [106, 107]. Rising Epac genes edited by CRISPRa can elevate Rap1 activity and impede cerebral injury and inflammation [28]. While early-phase trials for AAV-based therapies in HD and MS are ongoing, Rap1-specific gene therapies remain at the preclinical stage. More research is needed to monitor the efficacy and safety of these novel approaches. It needs to address challenges such as vector delivery efficiency, long-term expression control, and immune responses during the clinical exploration of Rap1-modulated gene therapies.

## CONCLUSION

This review is the first to comprehensively summarize Rap1-mediated pathways in neurodegeneration and highlight its potential as a therapeutic target. We have demonstrated that during neurodegenerative diseases, activation of Rap1 is a critical endogenous neuroprotective mechanism, effectively restraining immune-inflammatory responses and prohibiting neuronal impairments. A mounting number of evidence illustrates that Rap1 exerts crucial roles in maintaining synaptic plasticity, neuronal survival, and mitochondrial function. Considering the strongly activated inflammatory response in neurodegenerative diseases, the Rap1 signaling networks can mediate the interplay of neurotransmitters, peptides, and chemokines between the NVUs and immune systems. Rap1 acts as an integrator, capable of equilibrating the immune microenvironment and protecting neuronal activities. We analyzed the complexity of Rap1 signaling networks, detailing its involvement with downstream Ras, Rho, and Rac cascades, and their association with key pathways, including PI3K/Akt, MAPK/ERK, AMPK, and NF-κB. Furthermore, we elaborated on specific regulators, including endogenous gene modification, exogenous drugs, and natural medicines, to identify possible mechanisms and therapeutic effects. The accumulating preclinical evidence has proven Rap1 as a compelling integrator of key neurodegenerative pathways, drawing focused investigation in translational research programs.

In conclusion, Rap1 has attracted great attention not only as a molecular integrator of neuroprotective and anti-inflammatory pathways, but also as a highly promising therapeutic target. Further research is urgently needed to fully elucidate the complex regulating mechanisms and distinguish the specific, distinct roles of its isoforms, such as Rap1A and Rap1B. Targeted studies focusing on drug development and gene-based interventions will be essential in translating Rap1 modulation into novel and effective treatments for neurodegenerative diseases.

## Figures and Tables

**Fig. (1):**
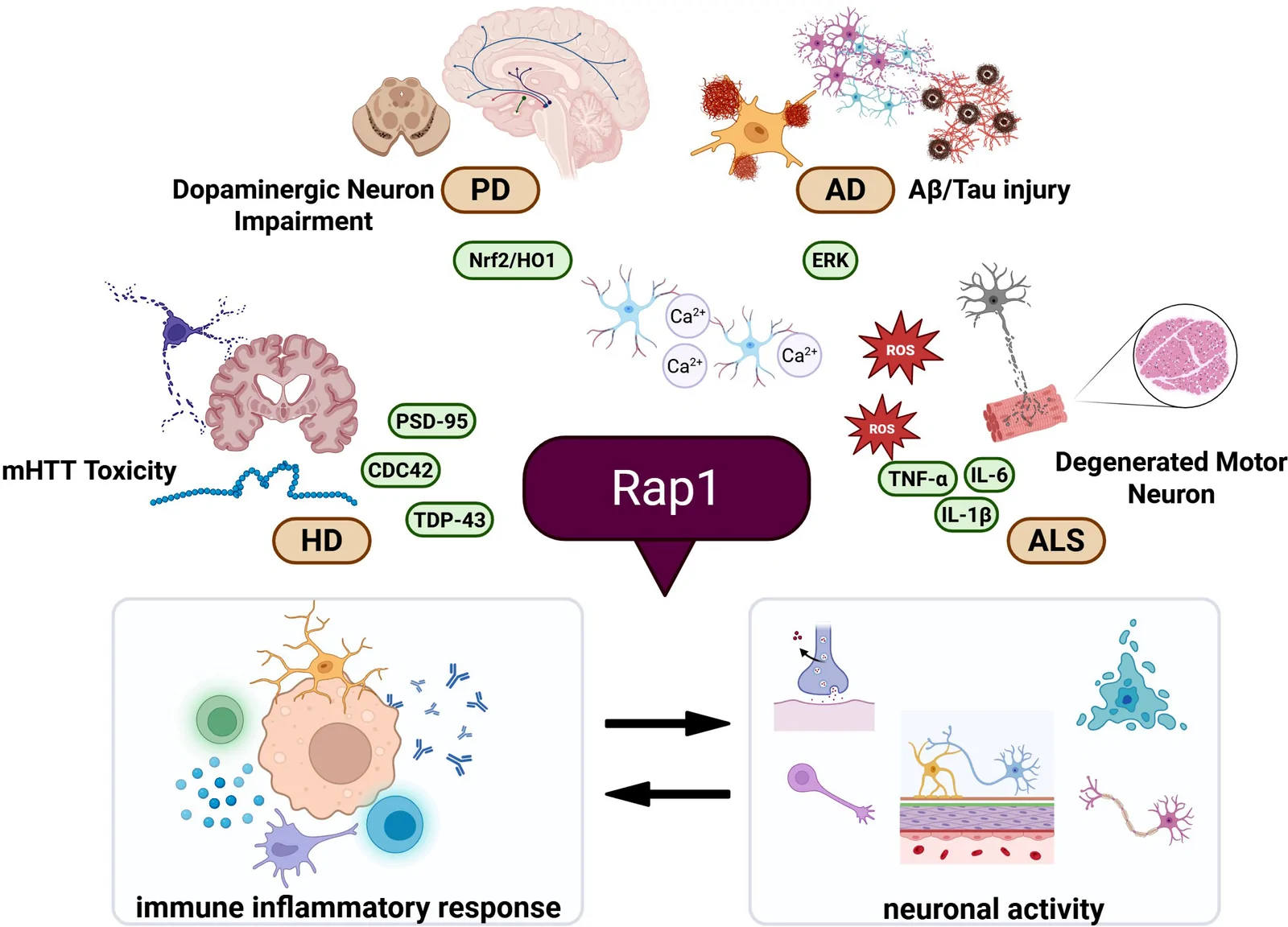
Rap1 regulates pathophysiological progression in neurodegenerative diseases.

**Table 1:** Emerging potential of Rap1 in neurological disorders.

**Diseases**	**Pathophysiology**	**Mechanism of Rap1**	**Effectors**	**References**
AD	Aβ plaques, Tau tangles	Recess neuroinflammation, neuroprotection, protect synaptic plasticity	Bax/Bcl-2, AMPA, NMDA, B-Raf, PI3K/Akt, ERK/MAPK, NF-κB	[48-59]
PD	Dopaminergic neuron degeneration, α-synuclein aggregation	Regulate the immune microenvironment, maintain mitochondrial function and autophagy, and inhibit neuronal apoptosis	BDNF, cAMP, PKA, AMPK, PI3K/Akt, ERK1/2	[60-68]
ALS	Motor neuron degeneration, activated microglia	Reduce neuroinflammation and oxidative stress, facilitate motor neuron survival	TNF-α, IL-1β, IL-6, BDNF, GDNF, NF-κB, Akt/ERK, PI3K/Akt, PKA/CREB	[48, 56, 69-79]
HD	mHTT aggregation, CAG repeat expansion	Inhibit synaptic dysfunction, regulate mHTT toxicity and autophagic flux	PSD-95, PGC-1α, CBP, B-Raf, ERK, cAMP, CREB, p38/MAPK	[28, 56, 80-88]
MS	Demyelination, glial activation	Modulates immune cell activation and neuronal survival	Epac, cAMP, ERK, AMPA, IL-10	[89-92,56,93]
Stroke	Ischemia-reperfusion injury	Reduce oxidative stress, inhibit neuronal apoptosis, protect the blood-brain barrier	VE-cadherin, claudin-5, afadin, Rac1, MMP, AMPA, NMDA, VEGF	[25, 94-98]

**Abbreviations:** AD: Alzheimer's disease, PD: Parkinson's disease, ALS: amyotrophic lateral sclerosis, HD: Huntington's disease, MS: multiple sclerosis, MAPK: mitogen-activated protein kinase, BDNF: brain-derived neurotrophic factor, GDNF: glial cell line-derived neurotrophic factor, cAMP: cyclic adenosine monophosphate, AMPA: α-amino-3-hydroxy-5-methyl-4-isoxazolepropionic acid, ERK: extracellular signal-regulated kinase, CREB: cAMP response element binding protein, PKA: protein kinase A, MMP: matrix metalloproteinase, NMDA: N-methyl-D-aspartate, VEGFA: vascular endothelial growth factor.

**Table 2:** Characteristics and mechanism of medicines activating Rap1.

**-**	**Compounds/Composition**	**Mechanism**	**Experimental Models**	**References**
**Drugs**				
Tirzepatide	GIP	Remit ferroptosis and keep neuronal homeostasis	NB41A3 cells, GIP-overexpressing transgenic mice	[86]
Deltarasin	Pde6δ	Decrease neuronal loss	hTAU-P301L cells, FVB/NxC57BL6 J	[48]
8-CPT	8-pCPT-2’-O-Me-cAMP	Reduce neuroinflammation and stabilize the blood-brain barrier	MCAO mice	[29]
**Natural Medicines**				
Lingguizhugan decoction	*Poria cocos* Schw. Wolf, *Cinnamomum cassia* Presl, *Atractylodes macrocephala* Koidz., and *Glycyrrhiza uralensis* Fisch. (4:3:3:2)	Downregulate levels of iNOS, TNF-α, and IL-1β, maintain neuronal viability	Aβ_1-42_-injected AD rats	[87]
*Rosmarinus officinalis* L	Genkwanin	Inhibit neuronal apoptosis, increase cellular activity	Aβ_25–35_-induced HT22 cells	[51]
Phytocannabinoids	Cannabidiol	Recess dopaminergic synaptic injury and neuroinflammation	Transgenic *C. elegans* OW13 PD model, 6-OHDA-injected PD rats	[88]
Sanghuang	Protocatechuic acid	Modulated platelet activity and endothelial inflammatory response	Vascular endothelia injured patients	[89]
Piper betle	Hydroxychavicol	Maintain synaptic plasticity and neuronal excitability	Human genes	[90]
Marine polyphenols	BK	Reduce neuroinflammation and enhance endothelial adhesion	Human genes	[91]
Leech	Neurohemerythrin, Hirudin, and Eglin C	Oxidative phosphorylation	Quantitative proteomics	[92]
**Gene modification**				
Rap1-CA	AAV	Reduce neuronal apoptosis and improve synaptic function	NIH3T3 cells, medium spiny neurons of the nucleus accumbens	[106, 107]
Epac genes	AAV- CRISPRa	Elevate Rap1 activity and reduce neuroinflammation	S2R+ cells	[28]

**Abbreviations:** BK: 8,8’-bieckol, GIP: glucose-dependent insulinotropic polypeptide, MCAO: middle cerebral artery occlusion, 6-OHDA: 6-hydroxydopamine, Rap1-CA: constitutively active Rap1, AAV: adeno-associated virus, CRISPR: clustered regularly interspaced short palindromic repeats.

## LIST OF ABBREVIATIONS

6-OHDA: 6-hydroxydopamine
AAV: Adeno-Associated Virus
AD: Alzheimer's Disease
ALS: Amyotrophic Lateral Sclerosis
AMPA: α-amino-3-hydroxy-5-methyl-4-isoxazolepropionic Acid
APP: Amyloid Precursor Protein
BDNF: Brain-derived Neurotrophic Factor
BK: 8,8’-bieckol
BMP: Bone Morphogenetic Protein
CAG: Cytosine-Adenine-Guanine
cAMP: Cyclic Adenosine Monophosphate
CBP: CREB-Binding Protein
CDC25: Cell Division Cycle 25
CNR1: Cannabinoid Receptor 1
CNS: Central Nervous System
CREB: cAMP Response Element Binding Protein
CRISPR: Clustered Regularly Interspaced Short Palindromic Repeats
EPSCs: Excitatory Postsynaptic Currents
ERK: Extracellular Signal-regulated Kinase
GAPs: GTPase-Activating Proteins
GDNF: Glial Cell Line-Derived Neurotrophic Factor
GEFs: Guanine Nucleotide Exchange Factors
GIP: Glucose-Dependent Insulinotropic Polypeptide
GTPase: Guanosine Triphosphatase
HD: Huntington's Disease
IL-6: Interleukin-6
IQGAP1: IQ Motif Containing GAP1
LTD: Long-Term Depression
LTP: Long-Term Potentiation
MAPK: Mitogen-activated Protein Kinase
MCAO: Middle Cerebral Artery Occlusion
METTL3: Methyltransferase-like 3
MMP: Matrix Metalloproteinase
NADPH: Nicotinamide Adenine Dinucleotide Phosphate
NLRP3: NOD-Like Receptor Family Pyrin domain-containing 3
NMDA: N-methyl-D-aspartate
PD: Parkinson's Disease
PKA: Protein Kinase A
PSD-95: Postsynaptic Density Protein 95
RalGDS: Ral Guanine Nucleotide Dissociation Stimulator
Rap1: Ras-proximate-1
Rap1-CA: Constitutively Active Rap1
ROS: Reactive Oxygen Species
SHANK2: SH3 and Multiple Ankyrin Repeat Domains 2
SOD1: Superoxide Dismutase 1
VEGFA: Vascular Endothelial Growth Factor A
